# Evaluating generative AI models for explainable pathological feature extraction in lung adenocarcinoma: grading assessment and prognostic model construction

**DOI:** 10.1097/JS9.0000000000002924

**Published:** 2025-07-09

**Authors:** Chong Lu, Jiadong Zhao, Wei He

**Affiliations:** a Department of Urology, The Four Affiliated Hospital of Anhui Medical University, Hefei, Anhui, P. R. China; b Shantou University Medical College, Shantou, Guangdong, P. R. China

*Dear Editor,*

We read with great interest the article by Shen *et al*, entitled “Evaluating generative AI models for explainable pathological feature extraction in lung adenocarcinoma: grading assessment and prognostic model construction”^[1,2]^. The authors present an ambitious and timely investigation into the application of generative AI (GenAI) models for histopathological grading and prognostic modeling in lung adenocarcinoma, a domain that remains at the frontier of computational pathology. While the study offers valuable insights, several methodological and conceptual limitations merit critical discussion.

First, the study design heavily relies on region-of-interest (ROI) selection, manually extracting three representative fields per slide at 10× magnification. While this approach simplifies computational processing, it potentially introduces significant selection bias. The exclusion of whole-slide imaging (WSI) analysis limits the representativeness of the assessed histologic patterns, especially in heterogeneous tumors where spatial distribution of patterns may substantially impact grading and prognostic assessment. This concern is well documented in prior studies emphasizing the superiority of WSI-based models in capturing tumor heterogeneity^[3]^. The authors acknowledge this limitation but do not sufficiently explore how manual ROI selection may distort model performance or generalizability. Future studies incorporating automated WSI analysis would better reflect the full complexity encountered in clinical practice.

Second, the evaluation of GenAI models largely emphasizes pattern recognition and grading consistency compared to human pathologists but lacks a thorough error analysis. Notably, all three GenAI models, including the best-performing Claude-3.5-Sonnet, demonstrate relatively limited accuracy in recognizing micropapillary patterns – one of the most clinically significant features in lung adenocarcinoma grading. Given that micropapillary components critically affect prognosis, insufficient recognition of such features raises concerns about the clinical readiness of these models. A detailed breakdown of misclassification rates across individual histologic patterns would have provided more granular insights into specific weaknesses of the evaluated models.

Third, although the prognostic model demonstrates reasonable discrimination with a C-index of 0.715, its incremental value beyond existing staging systems remains unclear. The inclusion of TNM stage, age, and sex as clinical variables may inherently drive much of the prognostic power, raising questions about how much unique prognostic information is truly added by GenAI-derived histologic features. Comparative analyses against conventional staging and grading systems would have strengthened the argument for integrating GenAI into routine clinical workflows.

Fourth, while the authors utilize multiple machine learning algorithms, the relatively small sample size for external validation (*n* = 95 and *n* = 87) may limit confidence in the generalizability of the proposed prognostic model. In particular, external validation set 2 failed to reach statistical significance (*P* = 0.112), which underscores the need for larger, more diverse multi-institutional datasets before clinical implementation can be seriously contemplated.

Fifth, the choice of evaluated GenAI models – GPT-4o, Claude-3.5-Sonnet, and Gemini-1.5-Pro – while justified based on current accessibility, may not fully reflect the performance of domain-specific vision foundation models trained explicitly on histopathology datasets. Several specialized models have demonstrated superior performance in digital pathology through large-scale pretraining on histology images, which general-purpose GenAI models currently lack^[4]^. A more balanced comparison, including dedicated pathology models (e.g., Panoptic, PathAI, or self-supervised WSI encoders), would offer a more comprehensive assessment of GenAI readiness for pathology applications.

Lastly, while the authors briefly mention the educational potential of GenAI in pathology training, the ethical and regulatory challenges inherent to AI adoption in clinical pathology – such as explainability, accountability, and data privacy – are not adequately addressed. As GenAI integration into diagnostic workflows progresses, robust governance frameworks will be essential to ensure safety, transparency, and clinician oversight^[5]^.

In conclusion, Shen *et al* present an innovative exploration of generative AI in lung adenocarcinoma pathology. However, technical limitations related to sample selection, pattern recognition accuracy, model interpretability, and external validity temper enthusiasm for immediate clinical translation. We encourage future research to address these gaps through whole-slide analyses, inclusion of larger multicenter datasets, and head-to-head comparisons with domain-specific models to further delineate the true clinical utility of generative AI in pathology.

## Data Availability

Data sharing is not applicable to this article as no new data were created or analyzed in this study.
